# Opportunities and Challenges to Profile mRNA Modifications in *Escherichia coli* **

**DOI:** 10.1002/cbic.202200270

**Published:** 2022-07-29

**Authors:** Dimitar Plamenov Petrov, Steffen Kaiser, Stefanie Kaiser, Kirsten Jung

**Affiliations:** ^1^ Department of Biology I, Microbiology Ludwig-Maximilians-University Munich Martinsried Germany; ^2^ Department of Chemistry Ludwig-Maximilians-University Munich Munich Germany; ^3^ Department of Pharmacy Goethe-University Frankfurt Frankfurt Germany

## Abstract

mRNA methylation is an important regulator of many physiological processes in eukaryotes but has not been studied in depth in prokaryotes. Working with bacterial mRNA is challenging because it lacks a poly(A)‐tail. However, methods for detecting RNA modifications, both sequencing and mass spectrometry, rely on efficient preparation of mRNA. Here, we compared size‐dependent separation by electrophoresis and rRNA depletion for enrichment of *Escherichia coli* mRNA. The purification success was monitored by qRT‐PCR and RNA sequencing. Neither method allowed complete removal of rRNA. Nevertheless, we were able to quantitatively analyze several modified nucleosides in the different RNA types. We found evidence for stress dependent RNA modification reprofiling in rRNA, but also several modified nucleosides in the mRNA enriched fractions showed significant changes.

## Graphical abstract

**mRNA methylation** is an important regulator in eukaryotes but has not been studied in depth in prokaryotes. In this study, we compared size‐dependent separation by electrophoresis and rRNA depletion for enrichment of *Escherichia coli* mRNA and quantitatively analyzed modified nucleosides in the different RNA types.

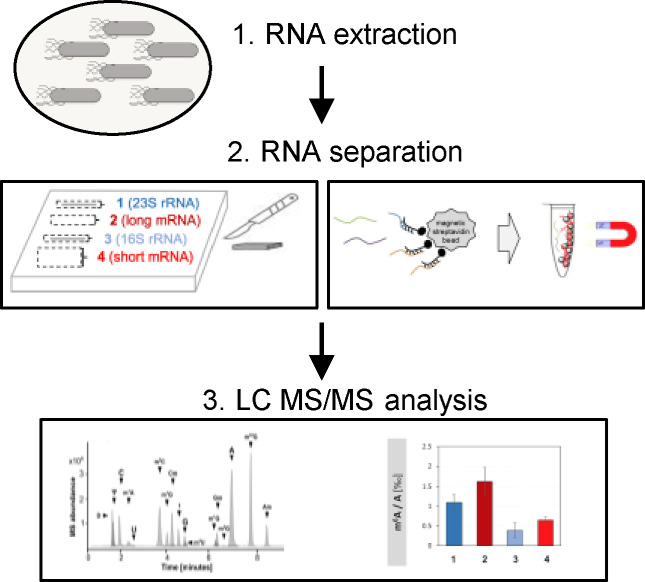

## Introduction

Chemical modification of DNA is a well‐known epigenetic regulatory mechanism. The recent development of highly sensitive analytical techniques has discovered increasing numbers of RNA modifications leading to new aspects of RNA regulation in the emerging field of epitranscriptomics. To date, more than 160 RNA modifications have been described in all domains of life.[^1^, ^2^] Eukaryotes carry both reversible and irreversible RNA modifications in their messenger RNA (mRNA). These mRNA modifications play an important role in RNA editing, splicing, and 5′‐capping, and some also have regulatory effects on RNA stability and translational fidelity.[^3^, ^4^]

In eukaryotes, tRNA and rRNA modifications can affect the whole protein synthesis in the cell, whereas several mRNA modifications have been shown to be posttranscriptional regulators of specific gene expression programs involved in cell development and homeostasis.[^5^, ^6^] *N* ^6^‐methyladenosine (m^6^A) is the most abundant mRNA modification in eukaryotes and is the best studied example of how mRNA methylation regulates both mRNA stability and gene expression.^[7]^ Pseudouridine (Ψ), one of the most abundant modifications found in all types of RNA, is also present in the coding sequence of mRNAs and has been reported to affect pre‐mRNA splicing,^[8]^ translational speed and tRNA selection by ribosomes.^[9]^ In general, studies of the epitranscriptome utilize sophisticated sequencing approaches which allow sequence‐specific localization of the RNA modification of interest. In contrast, mass spectrometric (MS) approaches of complete RNA hydrolysates reveal the full set of an RNA's chemical modification diversity but fail to reveal the sequence context.^[10]^

While bacterial tRNA and rRNA can be easily purified and the modification profile assessed through MS,[^11^, ^12^] knowledge regarding the mRNA modification profile of prokaryotes is extremely limited. MS analysis of bacterial 5’‐cap modifications is possible by using of cap specific enzymes,[^13^, ^14^] whereas detection and quantification of internal mRNA modifications is highly depending on the purity of the mRNA preparation. Working with bacterial mRNA is challenging because it lacks a poly(A)‐tail, which can be used for its enrichment,^[15]^ and its average half‐life time is very short (in the range of minutes).^[16]^ Despite these challenges, in 2015, high‐resolution transcriptome‐wide m^6^A profiling for the two bacterial model organisms *Escherichia coli* and *Pseudomonas aeruginosa* revealed both conserved and distinct distribution patterns of this modification.^[17]^ The m^6^A/A ratios observed (>0.2 %) were comparable to those of mammals (0.1–0.4 %) and meiotic yeast (0.25 %). Most m^6^A‐modified transcripts were associated with respiration, amino acid metabolism, and stress response, suggesting a potential regulatory role for mRNA methylation in bacteria. Therefore, it was suggested that RNA methylation might be involved in the adaptation of bacteria to different conditions and developmental stages, as has been demonstrated for eukaryotic cells.^[17]^ Unlike *E. coli* and *Pseudomonas* spp., two other Gram‐negative cyanobacteria (*Anabaena* sp. PCC 7120 and *Synechocystis* sp. PCC 6803) and representative species of Gram‐positive bacteria (*Staphylococcus aureus* and *Bacillus subtilis*) showed low m^6^A/A ratios (<0.04 % and <0.08 %, respectively).^[17]^ Finally, 5‐methylcytidine (m^5^C) has been mapped in the mRNA of the archaeon *Sulfolobus solfataricus*.^[18]^

Because m^6^A is the only described mRNA modification in bacteria, we were interested whether other modifications found in eukaryotes are also present in bacterial mRNA. To address this question, we isolated total RNA along the growth of *E. coli*, used two mRNA enrichment methods, electrophoretic separation by size and rRNA depletion, followed by stringent validation of RNA identity and analyzed the individual fractions by isotope dilution nucleoside mass spectrometry (LC‐MS/MS). Similar to a recent study in mice,^[19]^ our systematic study reveals the challenges connected to a size‐dependent purification of mRNA fractions but also highlights the benefits of an unbiased assessment of the chemical diversity of bacterial RNAs. We could identify RNA modifications which appear to be involved in bacterial stress response. These modifications were found in both the rRNA and the mRNA enriched fractions which prompts the question: Do bacteria utilize their RNA modifications as a stress response mechanism?

## Results and Discussion

### Profiling of RNA modifications in *E. coli* MG1655

**Figure 1:**
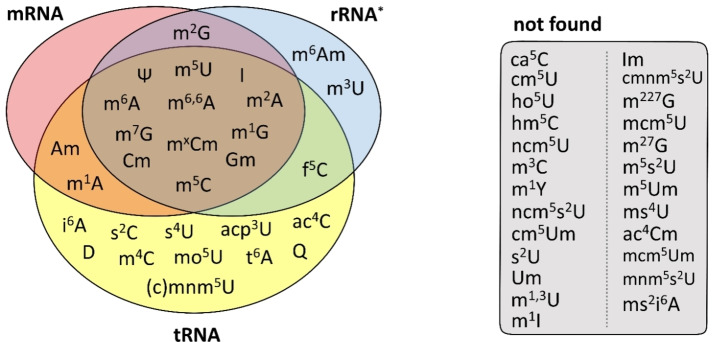
RNA modification profile of early log‐phase *E. coli* MG1655. Cells were aerobically cultivated in LB medium and harvested, and total RNA was isolated. The main RNA types were separated by size exclusion chromatography and subsequent rRNA depletion with commercial kits. After enzymatic hydrolysis, mass spectrometry was used to screen for 54 known modified nucleosides. 29 modified nucleosides were found in the mRNA, rRNA*, and tRNA fractions as indicated (left), and 25 were not found (right). * The results for rRNA represent the modifications found in the rRNA+mRNA fraction after tRNA removal by SEC, where the minor contribution of mRNA to the total modification levels is neglectable due to the excessive amounts of rRNA.

To investigate the complexity of mRNA modification in bacteria, we used the model organism *E. coli* MG1655. Since RNA sequencing is only possible for a limited number of modifications,[^20^, ^21^, ^22^] and negative controls are lacking because the modification machinery in bacteria is unknown, we used our established LC‐MS/MS‐based approach, which covers 54 known nucleosides, to assess the chemical diversity of the bacterial epitranscriptome. As a starting point, we performed qualitative profiling and comparison of the modifications in the three main RNA types: mRNA (including long noncoding RNA), rRNA (23S and 16S rRNA), and tRNA (including small RNAs less than 110 nucleotides (nt) in length). For this purpose, *E. coli* cells were grown in LB medium and harvested in the early exponential growth phase. Total RNA was isolated using phenol‐chloroform‐isoamyl alcohol (PCI) extraction and separated into tRNA and large RNAs containing both rRNA and mRNA (rRNA+mRNA fraction) by an established size exclusion chromatography (SEC) method.^[23]^ The successful removal of tRNA from the rRNA+mRNA fraction was verified by chip gel electrophoresis. We observed a clear distinction between the tRNA and rRNA+mRNA fractions (Figure S1A). As an additional control for the efficiency of tRNA separation, we measured m^6^A levels in the tRNA and rRNA+mRNA fractions in the *E. coli* wild‐type and the mutant ▵*trmM*, which lacks the only known methyltransferase for m^6^A methylation of tRNA.^[24]^ Whereas the m^6^A abundance in the rRNA+mRNA fraction remained similar in both strains, only substoichiometric amounts of m^6^A were detectable after LC‐MS/MS analysis in the tRNA fraction of the mutant, in contrast to a high level in the wild type, which shows efficient separation between the tRNA and rRNA+mRNA fractions (Figures S1B–C). Next, the purified rRNA+mRNA fraction was separated into rRNA and mRNA using a commercially available kit for the oligonucleotide‐based removal of rRNA. As commonly done to prove mRNA purity, we assessed the efficiency of rRNA depletion by RT‐qPCR, and for each mRNA sample, we observed at least a 500‐fold decrease in the rRNA amount compared to samples taken before oligonucleotide‐based rRNA depletion (Figure S2). Finally, each RNA fraction was enzymatically hydrolyzed, and the resulting nucleoside mixtures were subjected to LC‐MS/MS for qualitative analysis of 54 RNA modifications (Figure 1).

For each of the three studied RNA types – tRNA, rRNA, and mRNA‐enriched fraction – we observed a complex profile of modifications, as summarized in Figure 1. As expected, we found the highest chemical diversity in the tRNA fraction, with 26 different modified nucleosides detected. We confirmed numerous known tRNA modifications, and in addition, we detected *N* ^1^‐methyladenosine (m^1^A), m^6,6^A, 2′‐O‐methyladenosine (Am), and m^5^C in *E. coli* tRNA. The observation of m^6,6^A and m^5^C is in agreement with a recent report from the Helm lab, where small RNA fractions gained through size‐selection are contaminated with rRNA fragments and the latter has been identified as the source of rRNA specific modifications in mouse tRNA.^[19]^ As *E. coli* has no m^5^C reported in its tRNA, but in both rRNAs, we suggest that the m^5^C signal might be also rRNA fragment derived. The m^1^A signal might be related to a direct methylation of adenosine in tRNA upon exposure to natural methylating agents.^[25]^ While Am signals in small RNAs of mice are most likely caused by rRNA fragment contamination, the absence of Am in *E. coli* rRNA renders this potential origin as unlikely. Thus, it is likely that Am is a genuine modification of *E. coli* small RNA.

In the SEC‐purified rRNA fraction,^[23]^ we detected 16 modified nucleosides. Three of these modifications, namely, *N* ^6^, 2′‐O‐dimethyladenosine (m^6^Am), 5‐formylcytidine (f^5^C), and inosine, have not been observed previously in the rRNA of *E. coli*.^[11]^ A lower chemical diversity was observed in the mRNA fraction. Here, we detected 15 nucleosides overlapping with either tRNA or rRNA. Along with the only known modification m^6^A, in the mRNA of *E. coli* MG1655, we identified the presence of m^1^A, m^2^A, m^6,6^A, Am, m^5^C, 2′‐O‐methylcytidine (Cm), m^1^G, m^2^G, m^7^G, 2′‐O‐methylguanosine (Gm), m^5^U, inosine (I), and Ψ. In addition, we detected a modified cytidine derivative that is modified at both the ribose and the nucleobase (m^x^Cm). According to the literature, this modification is most likely N4,2’‐O‐dimethylcytidine (m^4^Cm), a reported 16S rRNA modification. Unfortunately, comparison with synthetic standards of m^4^Cm and m^5^Cm revealed co‐elution in our system, and so the existence of m^5^Cm cannot be excluded. Taken together, the modifications m^2^A, m^6^A, m^6,6^A, m^5^C, m^x^Cm, Cm, m^1^G, m^7^G, Gm, m^5^U, I, and Ψ were detected in all three types of RNA in *E. coli* MG1655 (Figure 1). Furthermore, m^2^G was detectable in the rRNA and mRNA‐enriched fractions, but not in the tRNA fraction, whereas m^1^A and Am were found in the tRNA and mRNA‐enriched fractions but not in the rRNA. The modifications m^6^Am and m^3^U were detected exclusively within the rRNA fraction. However, owing to their low abundance, they cannot be used as internal controls for rRNA contamination in our mRNA fractions. *N* ^6^‐Threonylcarbamoyladenosine (t^6^A), 2‐thiocytidine (s^2^C), 4‐thiouridine (s^4^U), and dihydrouridine (D) were found only in the tRNA fraction and might be suitable indicators for contamination with tRNA. Under our test conditions, 25 modifications known from both prokaryotes and eukaryotes were below the limit of detection in our RNA samples (Figure 1).

### Preparation of samples for quantitative analysis of mRNA and rRNA modifications

Our qualitative profiling of modifications in the three major RNA types obtained by commonly used RNA purifications technologies revealed the presence of at least two potential rRNA modifications (m^6,6^A and m^5^C) in the mRNA preparation which is an indication of obscured mRNA purity. For a rigorous qualitative and quantitative assessment of mRNA modifications, we decided to use agarose gel electrophoresis‐based size separation of RNAs and validate the resulting fractions using RT‐qPCR and RNA sequencing.

**Figure 2:**
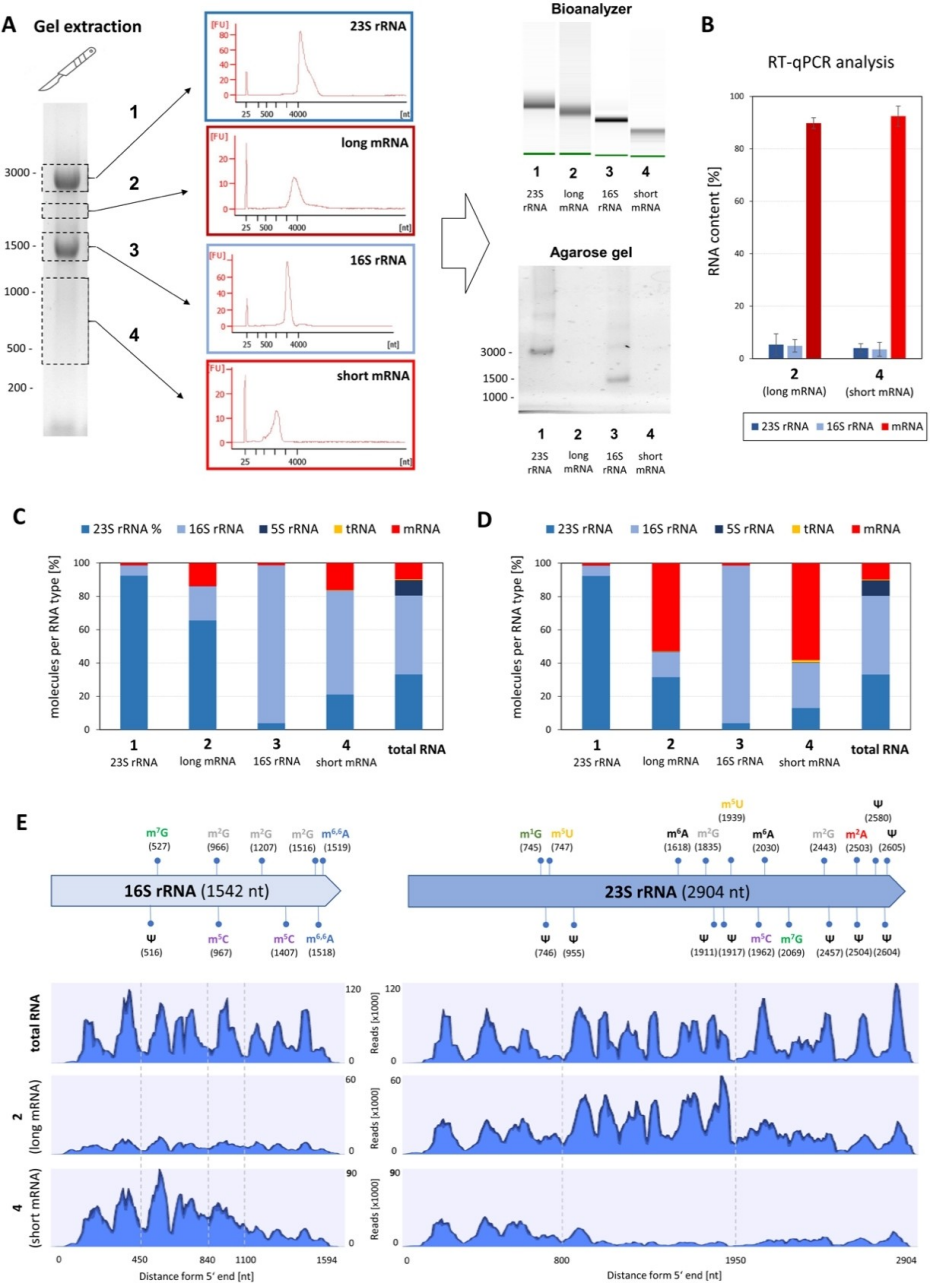
Analysis of RNA types from *E. coli* after separation by denaturing agarose gel electrophoresis. (A) After denaturing gel electrophoresis, total RNA was separated in four fractions: fraction 1 (23S rRNA), fraction 2 (mRNAs and noncoding RNAs of 1,700 to 2,700 bases), fraction 3 (16S rRNA) and fraction 4 (mRNAs and noncoding RNAs of 400 to 1,100 bases). The fractions were cut from the gel and after extraction analyzed by automated chip gel electrophoresis (Bioanalyzer, Agilent). The factions were reloaded onto an agarose gel to confirm the efficiency of separation. (B) Percentage of the major RNA types in the mRNA‐enriched fractions 2 and 4 determined by RT‐qPCR. Values were calculated based on an assumed ratio of 1 % mRNA and 90 % rRNA in the total RNA (see the Experimental Section for detailed description). (C) Molar ratios of major RNA types in the four fractions and the total RNA based on RNA‐Seq analysis. (D) Molar ratios of major RNA types found in the four fractions and the total RNA based on RNA‐Seq analysis after duplicate removal. (E) Schematic map of the localization of modifications in 23S rRNA and 16S rRNA of *E. coli*, and distribution of reads mapping to 23S rRNA and 16S rRNA sequences in samples of total RNA and fractions 2 and 4. B–D) Represent average results of three biological replicates with error bars of standard deviation from the means in B. E shows one representative example.

Total bacterial RNA was loaded on denaturing agarose gels, and after electrophoresis, four gel pieces per lane were excised. The RNA was extracted using a commercially available kit. According to size we obtained the following fractions: fraction 1 (23S rRNA), fraction 2 (mRNAs and noncoding RNAs of 1,700 to 2,700 bases), fraction 3 (16S rRNA) and fraction 4 (mRNAs and noncoding RNAs of 400 to 1,100 bases) (Figure 2A). The quality of each fraction was tested by chip gel electrophoresis and by running the extracted RNA on a denaturing agarose gel (Figure 2A). These controls indicated that we obtained efficient separation between the rRNA and mRNA fractions.

Next, we determined the ratio of mRNA : rRNA in the gel‐extracted samples by RT‐qPCR analysis using the two different internal calibrators *alaS* (2,631 nts) and *recA* (1,062 nts) for the long and short mRNA fractions, respectively. We observed at least a 1,000‐fold increase in the mRNA : rRNA ratio in both mRNA‐enriched fractions 2 and 4 when compared to the total RNA samples (Figures 2B and S3A). We got a similar result for mRNA samples obtained using the commercial oligonucleotides‐based kits, however, these kits appear to selectively deplete 16S rRNA while substantial amounts of 23S rRNA remain (Figure S2).

A second RT‐qPCR experiment focused on the possibility of a co‐purification of rRNA fragments in the mRNA‐containing fractions 2 and 4. We performed RT‐qPCR using primer pairs binding to three different regions of the rRNA sequences (5′‐, middle and 3′‐region). These results revealed that 16S rRNA and, to a lesser extent, 23S rRNA had been partially degraded at the 3′ end (Figure S3B). Based on a ratio of 1 % mRNA and 90 % rRNA in total *E. coli* RNA,[^26^, ^27^, ^28^] we determined a contamination of fractions 2 and 4 with approximately 10 % rRNA fragments (Figure S3C).

To further define the purity of our mRNA fractions 2 and 4, we subjected all four fractions as well as total RNA to deep sequencing (RNA‐Seq). As expected, fraction 1 (23S rRNA) and fraction 3 (16S rRNA) contained the expected rRNA, and 1.4 % and 1.3 % mRNA, respectively (Figure 2C). However, the RNA‐Seq results of the total read samples revealed an mRNA : rRNA ratio of only 14 : 86 for fraction 2 and 16 : 83 for fraction 4 (Figure 2C). It should be noted that during library preparation for RNA‐Seq, if the amount of starting material is small, the highly diverse mRNA targets may be amplified less efficiently than the homogeneous rRNAs. To reduce this potential PCR amplification bias, we applied the duplicate removal algorithm.[^18^, ^29^, ^30^] Taking this factor into account, our RNA‐Seq data indicated an mRNA : rRNA ratio of 53 : 47 for fraction 2 and 58 : 42 for fraction 4 (Figure 2D), which is still different from our RT‐qPCR data (Figure 2B). This result indicated that using RT‐qPCR as a quantitative measure of mRNA purity of a sample might bear the risk of method bias. Thus the usage of different, orthogonal methods for mRNA quality controls is important in order to analyze potential contaminations of the sample, affecting interpretation of the results.

Next, we mapped the reads of 16S and 23S rRNA in total RNA samples and mRNA fractions 2 and 4. As shown in Figure 2E, we find a different distribution. For example, reads mapping to the middle sequence of 23S rRNA (700–1900 nt) were overrepresented in fraction 2, whereas reads mapping to the 3′ sequence (1000–1542 nt) of 16S rRNA were underrepresented in fraction 4 (Figure 2E). This distribution and the localization of known modifications in 23S and 16S rRNA (Figure 2E) could be included in the assessment of which modifications were the result of contamination by rRNA fragments in the mRNA‐enriched fractions 2 and 4.

In summary, our quality controls revealed different degrees of contamination with rRNA of the mRNA‐enriched fractions 2 and 4, depending on the used method. Both RT‐qPCR and RNA‐Seq are semi‐quantitative technologies and therefore a clear statement on the degree of contamination is not possible. Both methods show a substantial enrichment of mRNAs in fractions 2 and 4, but depending on the data analysis used, an unfortunate high abundance of rRNA remains (Figures 2C and 2D).

### Quantitative analysis of modifications in mRNA‐enriched fractions after electrophoretic separation and rRNA depletion

**Figure 3:**
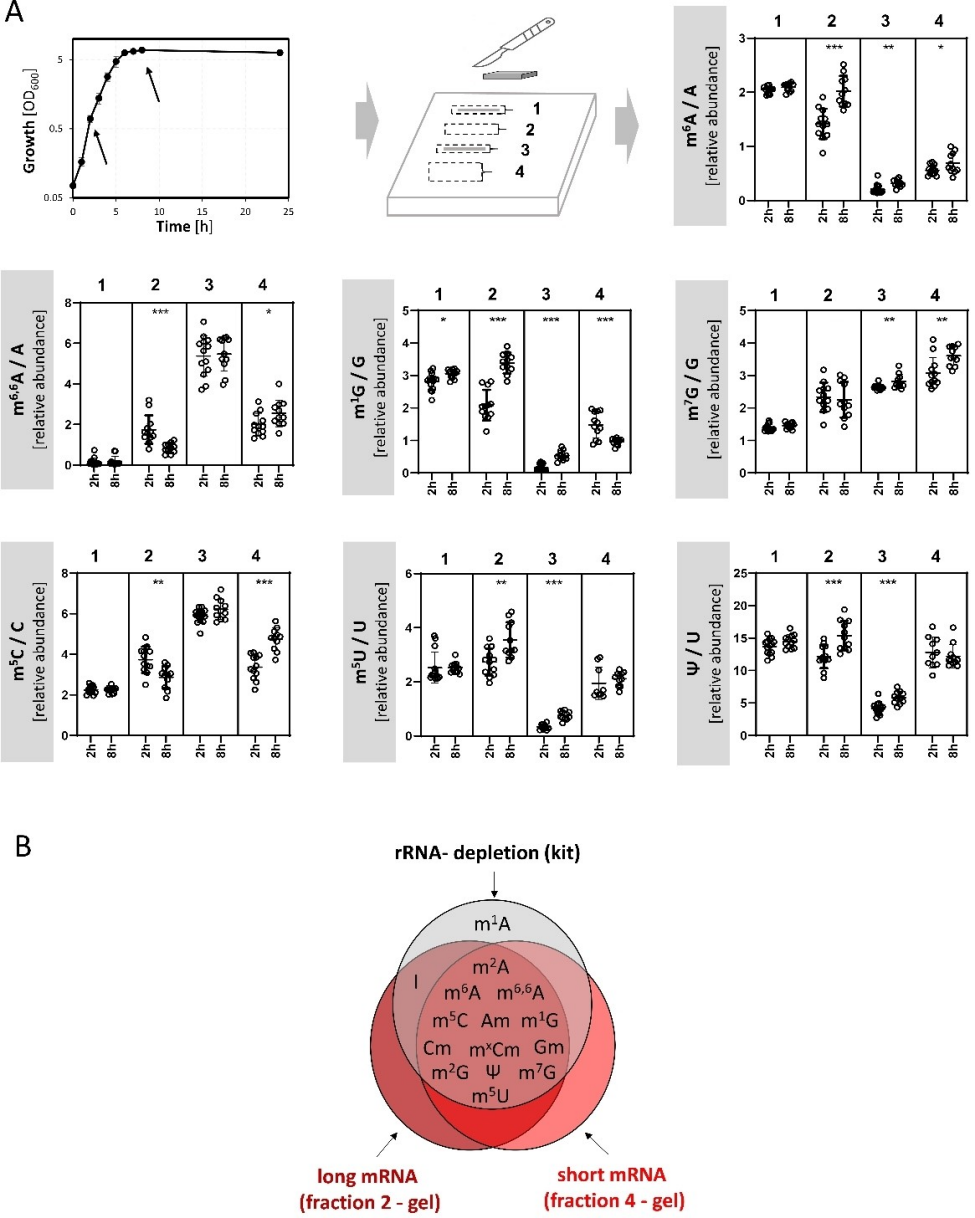
Quantitative analysis of modifications in RNA of *E. coli* after separation with denaturing gel electrophoresis and rRNA depletion. (A) RNA was isolated from *E. coli* MG1655 cells after cultivation in complex LB medium under aerobic conditions for 2 h (exponential growth phase) and 8 h (early stationary phase). Time points of RNA extraction are indicated by arrows in the growth curve. Following denaturing gel electrophoresis (see Figure 2) total RNA was separated in: fraction 1 (23S rRNA), fraction 2 (mRNAs and noncoding RNAs of 1,700 to 2,700 bases), fraction 3 (16S rRNA) and fraction 4 (mRNAs and noncoding RNAs of 400 to 1,100 bases). The fractions were cut from the gel and after rigorous quality controls, hydrolyzed and analyzed via LC‐MS/MS. The relative quantities of the modified nucleosides were normalized to their respective canonical precursors. The results of at least four biological replicates are presented, each measured in technical triplicates. p‐values were calculated using paired Student's t‐test with * ≤0.05, ** ≤0.01, and *** ≤0.001. (B) Comparison of the qualitative mRNA modification profiles after using two enrichment methods, the oligonucleotide‐based rRNA depletion and the denaturing gel electrophoresis.

We moved on to compare the separated RNA types with respect to the quantitative distribution of various modified nucleosides by LC‐MS/MS. We found m^6,6^A only in 16S rRNA, whereas m^6^A, m^1^G, m^5^U, and m^2^A were found in 23S rRNA (Figures 3 and S4B, Table 1), which is in agreement with the methylation patterns reported in previous studies (reviewed in Ref. [31]). Moreover, the stoichiometry of the modifications in the 23S and 16S rRNA fractions matched with the theoretical expectations based on the number of modification sites (Figures 2E and S4B). When calculating the expected abundance, we considered the fact that 23S rRNA is twice as long as 16S rRNA as the modification abundance would be inversely proportional to the length of the molecule. This effect was well demonstrated by the observed ∼40 % lower abundance of m^7^G in 23S rRNA (1 site: 1.38±0.11‰) compared to 16S rRNA (1 site: 2.63±0.07‰) (Table 1).

**Table 1:** Quantitative LC‐MS/MS analysis of the fractions 1–4 (Figure 3A), tRNA after SEC, and mRNA after rRNA depletion isolated from total RNA of *E. coli* MG1655. Cells were cultivated in LB medium for 2 h (exponential growth phase) or 8 h (stationary phase). The values represent the quantities of modified nucleosides after normalization to their respective canonical precursors (per 1,000 nucleosides). Results represent the mean and standard deviation of at least three biological replicates measured in technical triplicates. For comparison, theoretical values for modification frequencies in 16S and 23S rRNA are given, assuming that all known modification sites are modified.

Time	RNA type	m^6^A	m^2^A	m^6.6^A	m^1^G	m^7^G	m^5^C	m^5^U	Ψ
2 h	fraction 1 (23S rRNA)	1.85±0.42	1.33±0.13	0.16±0.19	2.81±0.27	1.38±0.11	2.16±0.35	2.52±0.57	13.71±1.21
8 h	fraction 1 (23S rRNA)	2.09±0.07	1.30±0.10	0.19±0.24	3.05±0.14	1.46±0.09	2.45±0.69	2.52±0.20	14.50±1.01
2 h	fraction 2 (long mRNA)	1.42±0.28	1.08±0.18	1.75±0.70	2.09±0.47	2.34±0.45	3.74±0.65	2.73±0.51	12.15±1.78
8 h	fraction 2 (long mRNA)	2.02±0.28	1.30±0.41	0.84±0.24	3.38±0.32	2.25±0.55	2.87±0.55	3.55±0.66	15.40±2.28
2 h	fraction 3 (16S rRNA)	0.19±0.09	0.10±0.03	5.32±0.97	0.14±0.11	2.63±0.07	5.82±0.35	0.33±0.10	4.07±0.91
8 h	fraction 3 (16S rRNA)	0.32±0.07	0.23±0.03	5.47±0.84	0.53±0.15	2.82±0.22	6.22±0.50	0.75±0.15	5.85±0.94
2 h	fraction 4 (short mRNA)	0.56±0.09	1.23±0.36	2.01±0.55	1.48±0.40	3.08±0.47	3.37±0.61	2.12±0.77	11.94±2.94
8 h	fraction 4 (short mRNA)	0.69±0.19	1.16±0.21	2.55±0.66	0.96±0.10	3.61±0.29	4.75±0.55	2.12±0.25	12.21±1.78
	23S rRNA (theoretical)	2.62	1.31	0	1.10	1.10	1.57	3.30	16.60
	16S rRNA (theoretical)	0	0	5.14	0	2.06	5.68	0	3.17
2 h	tRNA	1.69±0.20	NA	0.44±0.10	7.46±0.30	25.55±0.56	0.34±0.08	97.14±1.71	136.29±4.04
2 h	mRNA (rRNA depletion)	0.50±0.15	1.44±0.08	1.18±0.77	0.85±0.29	0.92±0.22	1.17±0.66	1.35±0.83	5.88±1.83

Quantitative LC‐MS/MS analysis of the gel‐extracted mRNA‐containing fractions 2 and 4 was consistent with our qualitative results. We detected m^6^A, m^6,6^A, m^1^G, m^7^G, m^5^C, m^5^U, and Ψ in both the long and short mRNA‐enriched fractions 2 and 4 in stoichiometrically relevant quantities using a stable isotope‐labeled internal standard from *Saccharomyces cerevisiae* tRNA (SILIS)^[32]^ (Table 1). m^2^A was also detected in the mRNA fractions at a similar abundance as in the 23S rRNA fraction (Figure S5). However, due to the absence of m^2^A in the yeast‐derived SILIS, further analysis and quantitative assessment of m^2^A was not possible.

Overall, our mRNA‐enriched fractions 2 and 4 showed a reproducible modification profile that is different from the 23S and 16S rRNA modification profiles and quantitatively decoupled (Figure 3A). For example, m^6^A is lower in fraction 2 compared to the preceding 23S rRNA fraction, whereas modifications such as m^7^G, m^5^C, m^5^U and Ψ are higher. In contrast, m^6^A, m^1^G, m^7^G, m^5^U and Ψ are higher in fraction 4 compared with the preceding 16S rRNA fraction, whereas m^5^C is lower and of similar abundance as in fraction 2.

The quantification results for fractions 2 and 4 showed that our measured m^6^A abundance (on average 1.1‰) is comparable to those published previously by Deng et al.^[14]^ Strikingly, modifications such as m^1^G, m^7^G, m^5^C, m^5^U, and Ψ had higher abundances in the mRNA‐enriched fractions 2 and 4 (Table 1). The most abundant modification in the mRNA‐enriched fractions 2 and 4 was Ψ (approximately 12‰). The m^5^C abundance detected in the fractions containing mRNAs was about 3.5‰. The abundance of m^6,6^A was comparable to that of m^6^A. For several modifications, we observed different methylation levels between the two mRNA‐enriched fractions. In fraction 2, containing long mRNAs, we measured a 2.5 times higher m^6^A abundance compared to fraction 4, containing short mRNAs. The abundance of m^1^G in fraction 2 was also higher than that in fraction 4. Conversely, for m^7^G, we observed a lower abundance in fraction 2 compared to that in fraction 4 (Figure 3A and Table 1). For other modifications such as m^5^C and Ψ, we did not observe significant differences between the long and short mRNA‐enriched fractions.

To validate the results for the RNA modification profile obtained by denaturing gel electrophoresis, we subjected the same total RNA to the established oligonucleotide‐based rRNA depletion protocol. Despite relying on different strategies, both approaches resulted in the qualitative identification of nearly identical RNA modifications after LC‐MS/MS analysis (Figure 3B). In addition to m^6^A, both approaches identified the presence of m^6,6^A, m^1^G, m^2^G, m^7^G, m^5^C, m^5^U, Am, Gm, Cm, m^x^Cm and Ψ in the mRNA‐enriched fractions from *E. coli*. Inosine (I), the deamination product of adenosine, known from a process called A‐to‐I mRNA editing, and a wobble base in tRNA,^[33]^ could also be a potential artefact derived from chemical adenosine deamination.It was only detected in the rRNA‐depletion approach and in the gel‐extracted long mRNA enriched fraction 2, but not in fraction 4.

When comparing the quantitative results of the two approaches, we generally observed higher modification abundances in the gel‐extracted samples compared to those using the rRNA depletion approach (Figure S4A). Nevertheless, the results of both approaches showed similar profiles: Ψ was the most abundant modification, followed by m^5^C, and m^5^U, whereas m^6^A was among the least abundant modifications found in the mRNA‐enriched fractions of *E. coli*. Importantly, the RNA profile of mRNA‐enriched samples was distinct from the tRNA as well as the 23S and 16S rRNA profiles of *E. coli* (Figure S4). The clearest difference between the profiles of the three RNA types was observed in the ratios between different modifications: m^6,6^A : m^5^C : m^5^U : m^7^G : Ψ ratio was 0 : 0 : 4 : 1 : 6 in tRNA, 0 : 2 : 3 : 1 : 15 in 23S rRNA, 5 : 5 : 0 : 2 : 3 in 16S rRNA, and 2 : 4 : 3 : 2 : 12 in mRNA‐enriched fractions (Figure S4). These ratios of RNA modifications are an additional indication that we were able to prepare substantially mRNA‐enriched fractions.

### Dynamics of mRNA modification

In eukaryotes, mRNA methylation is a dynamic, reversible process that has regulatory functions and involves a complex system of writers, readers, and erasers.[^7^, ^31^] Therefore, we were interested in whether the modifications of bacterial RNA change in relative abundance depending on the growth phase. We compared the modification patterns between the early exponential growth phase (2 h) and the stationary phase (8 h) (Figure 3A). We observed statistically significant increases in m^6^A, m^1^G, m^5^C, and Ψ, and a significant decrease in m^6,6^A in some of the mRNA‐enriched fractions of stationary phase cells compared with mRNA derived from the exponential growth phase cells. While for m^6^A the increase in modification abundance towards the stationary phase was observed in fractions 2 and 4, for m^1^G and Ψ, we observed an increase only in fraction 2, and for m^5^C only in fraction 4. Our chip gel analysis suggested no differences in rRNA integrity between RNA from cells in the exponential and early stationary phase (Figure S6).

In contrast, the abundances of rRNA modifications in *E. coli* between exponential and stationary phase cells were mostly unchanged, as shown by the semi‐quantitative analysis of fractions 1 and 3 (Figure 3A). We only measured a small increase in the abundance of m^6^A, m^1^G, and Ψ in the 16S rRNA‐containing fraction 3 of stationary phase cells. However, for example, m^6^A is not known to exist in 16S rRNA, and the changes in the measured amounts are similar to the changes observed for the mRNA‐enriched fractions 2 and 4. Therefore, it is likely that these results are a consequence of the co‐purification of mRNAs of the same size.

**Figure 4:**
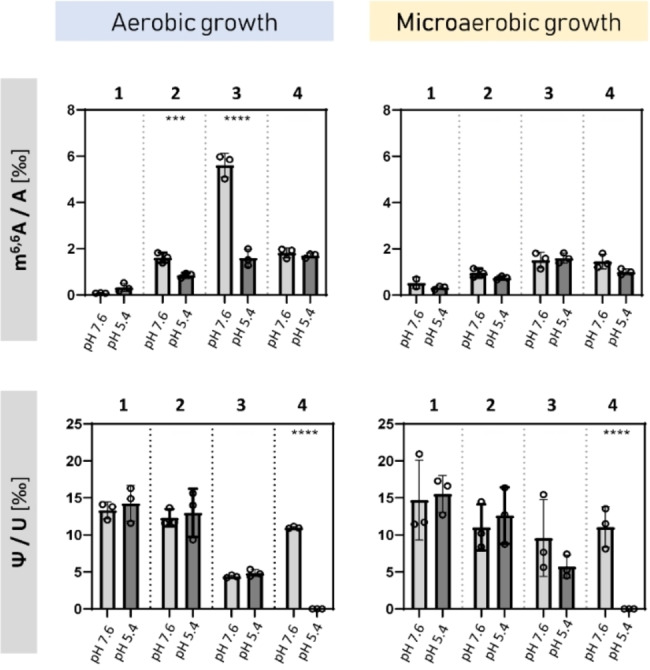
Impact of oxygen limitation and acid stress on the RNA modifications in *E. coli. E. coli* MG11655 was cultivated in complex LB medium at pH 7.6 or pH 5.4 under aerobic and microaerobic conditions. Total RNA was isolated and separated by denaturing gel electrophoresis (see Figure 2) in: fraction 1 (23S rRNA), fraction 2 (mRNAs and noncoding RNAs of 1,700 to 2,700 bases), fraction 3 (16S rRNA) and fraction 4 (mRNAs and noncoding RNAs of 400 to 1,100 bases). The fractions were cut from the gel and after rigorous quality controls, hydrolyzed and analyzed via LC‐MS/MS. The relative quantities of the modified nucleosides were normalized to their respective canonical precursors. The results of three biological replicates are presented. P‐values were calculated by paired Student's t‐test with * ≤0.05, ** ≤0.01, and *** ≤0.001.

Next, we tested whether the modification profile of mRNA in *E. coli* changes under the influence of external factors such as pH or oxygen availability. We cultivated cells in tryptone‐based (LB) medium at pH 7.6 or buffered at pH 5.4 under aerobic or microaerobic (oxygen limited) conditions (Figure 4 and Figure S7). Changes of the mRNA modifications observed during the transition from exponential growth to stationary phase under aerobic conditions (Figure 3A) were also found under oxygen limiting conditions (Figure S7). Specifically, we observed a statistically significant increase in m^6^A, m^1^G, and m^5^U and a decrease in m^5^C in the long mRNA‐enriched fraction 4 of stationary phase cells both under aerobic and microaerobic conditions.

However, we noted a sharp decrease for the m^6,6^A levels in 16S rRNA upon oxygen limitation. During aerobic growth the m^6,6^A abundance in fraction 3 (16S rRNA) was 5.8±0.35‰ (Figure 3A), and under microaerobic conditions there was a more than threefold reduction to 1.51±0.35‰ (Figure S7).

Comparison of cells cultivated under physiological condition (pH 7.6) and acid stress (pH 5.4) revealed stress‐specific changes for two modifications: m^6,6^A and Ψ (Figure 4). The cultivation of *E. coli* at pH 5.4 led to a threefold reduction in the m^6,6^A abundance in fraction 3 (16S rRNA) and to a smaller extent in fraction 2 (long mRNA‐containing fraction). An even more dramatic effect was observed for Ψ. Cultivation of *E. coli* under aerobic or microaerobic conditions at pH 5.4 resulted in a loss of Ψ in fraction 4 (Figure 4). This effect was target‐specific and not caused by a general issue with RNA as the levels of other modifications in this fraction were not affected (data not shown). In addition, the Ψ level of fraction 2 was not significantly altered by acid stress. These results indicate a correlation between environmental stress adaptation and rRNA modification in *E. coli*.

## Conclusion

Recent advances in the analysis of RNA modifications by sequencing and mass spectrometry have led to an ever‐rising number of studies focusing on the molecular mechanism of mRNA modifications in eukaryotes. Systematic and careful studies have also revealed the potential pitfalls and provided guidelines for avoiding them.[^19^, ^34^, ^35^, ^36^] For prokaryotes, the number of studies is currently small, but given the potential of the epitranscriptome for bacterial fitness, stress adaptation and thus human health in the context of bacterial infection, more studies will follow. Therefore, we set out to systematically test different methods for mRNA preparation using the model bacterium *E. coli* MG1655.

Agarose gel electrophoresis has been used previously for secondary purification of eukaryotic mRNA.[^37^, ^38^] Here we used and validated this simple and low‐cost method to overcome some of the existing difficulties. One of the main advantages of our electrophoretic approach is the direct, simultaneous separation of different RNA types, which allows a comparison of RNA fractions that underwent identical chemical treatment. This avoids potential bias due to different separation methods for each RNA type, primer selection, or low antibody specificity and allows direct comparison between fractions and thus using known modification patterns of tRNA and rRNA as internal controls. However, from our data, it appears that even under the most stringent conditions, a complete removal of rRNA and its various fragments has not yet been possible. Furthermore, our study indicates that more than one quality control, such as RNA‐Seq and RT‐qPCR, is necessary to estimate the level of rRNA contamination in mRNA‐enriched samples.

Our approach allowed us to analyze the chemical diversity of different RNA types subjected to identical treatment within a single analytical experiment. While our chosen LC‐MS/MS approach does not allow sequence specific localization of RNA modifications and is highly dependent on the purity of the input RNA,^[19]^ it is the only way to unambiguously determine the complete chemical RNA modification landscape in a sample and to identify the most interesting modifications of an organism's epitranscriptome before turning to laborious and expensive RNA‐modification sequencing technologies.[^39^, ^40^] The simultaneous measurement of multiple modifications allowed the creation of modification profiles (Figure S3, Table 1) enabling the assessment of possible contamination with tRNA. For example, the m^6,6^A : m^5^C : m^5^U : m^7^G : Ψ ratio of tRNA differed substantially from the ratio in the mRNA‐enriched fractions. Furthermore, the LC‐MS/MS approach is of highest sensitivity and thus ng amounts of RNA are sufficient for analysis. In contrast, direct RNA sequencing using Nanopore Sequencing requires about 500 ng of mRNA.^[41]^ Such amounts are difficult to obtain for bacterial mRNA due to the lack of poly(A)‐tails and enrichment technologies based on it.

Future studies need to focus on mapping modification sites in bacterial mRNA and the dynamics of the bacterial epitranscriptome. Due to the low degree of homology, knowledge about mRNA‐modifying enzymes is not yet available for prokaryotes. Since transcription and translation occur in the same compartment in prokaryotic cells, it would not be surprising if some RNA methyltransferases could modify more than one type of RNA.[^42^, ^43^, ^44^, ^45^]

In *E. coli* the GTPase MnmE plays an essential tRNA modifying function under acid stress, but the exact mechanism is still unclear.^[46]^ In yeast, stress‐dependent adaptation of tRNA modifications has been observed^[47]^ and attributed to halted transcription of tRNAs.^[48]^ Here, we observe an adaptation of RNA modifications in response to oxygen availability and acid stress. The functional significance of the absolute loss of pseudouridine under acid stress in fraction 4 is currently unclear, but we hypothesize that the adaptation of pseudouridine content is related to the bacterial acid stress response. Future studies including identification of the responsible pseudouridine synthases are needed to elucidate the importance of bacterial RNA pseudouridylation.

Taken together, our study systematically addresses the challenges of mRNA purification in bacteria, but also demonstrates the benefits of an unbiased assessment of bacterial RNA modification profiles through LC‐MS/MS analysis. Our data give us confidence that RNA modification is not a specific regulatory feature of eukaryotes but also appears to have a regulatory function in prokaryotes. In bacteria that exhibit rapid mRNA turnover, alteration of subsets of functionally related mRNAs with different modifications that specifically affect mRNA stability or translation may be an efficient way to prioritize certain cellular programs.

## Experimental Section

### Strains and growth conditions

*E. coli* K‐12 MG1655 and *E. coli* BW25113 ▵*trmN* carrying a chromosomal deletion of *trmN* ^[49]^ were used. All strains were grown in LB medium (10 g/L tryptone, 5 g/L yeast extract, and 10 g/L NaCl, pH 7.6) overnight. Bacteria were grown under agitation (200 rpm) at 37 °C, and growth was monitored over time by measuring the optical density at 600 nm (OD_600_). Cells from the overnight culture were used to inoculate fresh LB medium or LB‐MES medium (LB plus 0.1 M MES, pH 5.4). Bacteria were grown under agitation (200 rpm) at 37 °C, and growth was monitored over time by measuring the optical density at 600 nm (OD_600_). For microaerobic conditions, the cells were cultivated without shaking in closed, full vessels without access to air (Schott Duran bottles GL45, Jena, Germany).

### Total RNA purification

RNA was isolated using the PCI protocol^[50]^ with modifications. Cell pellets corresponding to 0.5 g (wet weight) were washed in 1 mL of ice‐cold AE buffer (20 mM sodium acetate buffer, pH 5.2, 1 mM EDTA) and resuspended in 500 μL of the same buffer. Then, 500 μL of pre‐warmed PCI for RNA extraction (Carl Roth, Karlsruhe, Germany) and 25 μL of 10 % (w/v) SDS were added, and the mixture was incubated for 5 min at 60 °C under vigorous agitation. Samples were cooled on ice for 2 h and centrifuged at 16,000×*g* for 1 h. The supernatant was transferred to phase‐lock tubes (Phase Lock Gel, QuantaBio, USA), and 1.0 volume of PCI and 0.1 volume of 3 M sodium acetate (pH 5.2) were added before centrifugation for 15 min. The supernatant was collected, mixed with 2.3 volumes of ethanol, and placed in a freezer at −80 °C overnight. After centrifugation at 16,000×*g* for 1 h, the supernatant was discarded, and the pellet was washed twice with 70 % (v/v) ethanol, dried, and resuspended in 100 μL of RNase‐free water. After treatment of the samples with RNase‐free DNase (Roche) according to the manufacturer's protocol, the samples were purified with another round of precipitation. The integrity of RNA was assessed by chip gel electrophoresis (Bioanalyzer 2100, RNA Nano chip kit, Agilent, Waldbronn). To evaluate the extent of DNA contaminations, the RNA preparation samples were tested by spectroscopic measurements (Nanodrop One) and qPCR.

### RNA separation with size exclusion chromatography

The separation of tRNA by size exclusion chromatography (SEC) was performed as described previously^[51]^ with minor modifications. Briefly, total RNA was loaded on a Thermo Scientific UltiMate 3000 LC system equipped with a diode array detector set to 260 nm, an autosampler, a column thermostat (60 °C), and a fraction collector. A size exclusion column (Agilent Bio SEC‐3, 3 μm, 300 Å, 7.8×300 mm, Agilent, Waldbronn) allowed the collection of the RNA fractions after isocratic elution with 100 mM ammonium acetate at pH 7. The peaks representing the mRNA+rRNA fraction as well as the tRNA fraction were collected and concentrated in a vacuum concentrator (Eppendorf Concentrator 5301). 5 M NH_4_OAc was added to a final concentration of 0.5 M, and after addition of 2× Vol. ice‐cold ethanol (100 %), the RNA was precipitated at −20 °C overnight. After centrifugation at 12,000×*g* for 30 min at 4 °C, the RNA pellet was subjected to an additional ethanol (80 %, v/v) wash step to verify the complete removal of the ammonium acetate and was then resuspended in pure water. The quality of the isolated tRNA was verified with chip gel electrophoresis (BioAnalyzer 2100, RNA Pico chip, Agilent, Waldbronn), RT‐qPCR analysis, agarose gel electrophoresis, and usage of the ▵*trmN* deletion mutant. RNA concentration was determined by NanoDrop ND1000 spectrophotometer (peqlab, Germany).

### Gel electrophoresis and RNA extraction

For gel electrophoretic extraction of RNA, denaturing 1.2 % (w/v) agarose gels (agarose – Serva, Germany), 20 mM MOPS, 1.1 % (v/v) formaldehyde (dissolved in DEPC‐treated water) were used. Before loading 10 μg of RNA, each sample was mixed 1 : 1 with sample buffer [64 % (v/v) desalted formamide, 8.35 % (v/v) formaldehyde, 26 mM MOPS, and 0.05 % (v/v) ethidium bromide]. After the addition of 1/10 volume RNA‐marker [50 % (v/v) glycerol, 1 mM EDTA, bromophenol blue, and xylene cyanol], the samples were denatured at 65 °C for 5 min, followed by immediate cooling on ice. The gels were run in running chambers (Bio‐Rad) using 20 mM MOPS as a running buffer at 5 V/cm for 3 h. As a size reference, the RiboRuler high range RNA ladder (Thermo Scientific) was used. RNA fragments with sizes from 3,200 to 2,800 nt (23S rRNA), 2,700 to 1,700 nt (long mRNAs), 1,600 to 1,400 nt (16S rRNA), and 1,100 to 400 nt (short mRNAs) were cut from the gel, leaving physical space between each of them. Cutting was documented before and after the excision of the fragments. RNA was isolated from the gel fragments with a commercially available kit (Hi Yield Gel/PCR DNA Fragment Extraction Kits, Suedlabor Gauting, Germany). Elution from the column was performed in two consecutive steps using 25 μL water. The quality of each fraction was analyzed using chip gel electrophoresis (Bioanalyzer 2100, RNA Pico chip kit, Agilent, Waldbronn) and another denaturing agarose gel electrophoresis. The efficiency of RNA separation was tested using Bioanalyzer chip electrophoresis and RT‐qPCR.

### Oligonucleotide‐based depletion of rRNA

After removal of tRNA and small noncoding RNAs by SEC, the remaining mRNA+rRNA mixture was subjected to two consecutive rounds of rRNA depletion according to the manufacturer's protocol for oligonucleotide‐based depletion of rRNA (RiboPOOL oligo pool for *E. coli*, siTOOLS, Martinsried, Germany). The efficiency of rRNA depletion was tested by Bioanalyzer chip electrophoresis and RT‐qPCR.

### RT‐qPCR analysis

For RT‐qPCR analysis, equal amounts of the isolated total RNA or the isolated RNA fractions were converted to cDNA with the iScript Advanced Script (Bio‐Rad) according to the manufacturer's protocol. The samples were mixed with SsoAdvanced Univ SYBR Green Supermix (Bio‐Rad), dispensed in triplicate into a 96‐well PCR plate (Bio‐Rad), and subjected to qPCR in a Bio‐Rad CFX real‐time cycler (see Table S1 for primers). For internal calibration, the data were evaluated using the ▵▵*C*_T_ method^[52]^ using rRNA and *recA* and *alaS*.

### Analysis of mRNA enrichment by RT‐qPCR

To evaluate the level of RNA separation and mRNA enrichment in the mRNA fractions extracted after denaturing gel electrophoresis (fractions 2 and 4) and after oligonucleotide‐based rRNA depletion, the ratios of mRNA : 16S rRNA and mRNA : 23S rRNA were determined by RT‐qPCR. To calculate the level of rRNA removal, the mRNA : rRNA ratios before (total RNA or the mRNA+rRNA fraction) and after gel electrophoresis or oligonucleotide‐based rRNA depletion (mRNA fractions) were compared. The ▵▵*C*_T_ method^[52]^ was used to minimize potential errors due to imprecise determination of the RNA concentrations of the total RNA and mRNA samples. 16S and 23S rRNA were regarded as “genes of interest” and mRNA targets as “reference genes” for internal calibration (the reverse scenario of a standard calculation of gene expression). *alaS* (2,631 nts) was used as internal reference for the long mRNA fractions and *recA* (1,062 nts) for the short mRNA fractions and the mRNA after oligo‐based rRNA depletion with RiboPOOL.

To calculate the mRNA : rRNA ratios as percentage, a ratio of 90 % rRNA (45 % 23S rRNA, 45 % 16S rRNA) and 1 % mRNA in the total RNA[^26^, ^27^, ^28^] was assumed resulting in 1 : 45 ratios of mRNA : 23S rRNA and mRNA : 16S rRNA. Then, the calculated fold‐changes for the *recA* : 16S rRNA and *alaS* : 23S rRNA ratios between the total RNA samples and the mRNA‐enriched fractions were used to calculate the final mRNA : rRNA ratios. For example, at a fold‐change of 90 between *alaS* : 23S rRNA, the mRNA : 23S rRNA ratio would be 2 : 1, and similarly, for a fold‐change of 90 between *recA* : 16S rRNA, the mRNA : 16S rRNA ratio would be 2 : 1. Taken together, the final ratio of mRNA : 23S rRNA : 16S rRNA would be 2 : 1 : 1 or 50 % mRNA and 50 % rRNA.

### Analysis of mRNA enrichment by RNA sequencing

To evaluate the level of contaminating rRNA in the mRNA‐enriched fractions 2 and 4, the RNA was converted into cDNA libraries using the NEBNext Ultra RNA Library Prep Kit for Illumina (New England Biolabs, USA). As a control, libraries were prepared from the corresponding total RNA. The libraries were analyzed in an HiSeq Illumina sequencer with an average sequencing depth of ∼8 million single‐end 50 nt‐long reads.

Data analysis was performed with the CLC Genomic workbench software (Qiagen, Germany) with or without duplicate removal, and both data sets were analyzed independently. To evaluate the molar ratios of RNA types in the samples, the quotient of (reads for a specific gene)/(total reads) was divided by the length of the specific gene.

### MS analysis of RNA methylation

Qualitative (no internal standard, no calibration), semi‐quantitative (internal standard, no calibration) and absolute quantitative analyses (internal standard and calibration) of the prepared RNA fractions were performed by LC‐MS/MS as previously described.^[32]^ After the enzymatic hydrolysis of the RNA to nucleosides using a mixture of benzonase, snake venom phosphodiesterase and calf intestine phosphatase,^[32]^ the improved gen^2 13^C/^15^N stable isotope labeled internal standard (SILIS) from *S. cerevisiae* tRNA was added^[53]^ for semi‐quantitative and absolute quantitative analysis. The resulting ribonucleoside mixture was separated using a Synergy Fusion RP column, with 2.5 μm particle size, 100 Å pore size, 100 mm length, and 2 mm inner diameter from Phenomenex (Torrance, CA, USA), on an Agilent 1290 Infinity II series UHPLC. Mobile phase A was 5 mM ammonium acetate adjusted to pH 5.3 with glacial acetic acid, and mobile phase B was pure acetonitrile. Gradient elution started with 100 % A for 1 min, increased to 10 % B after 4 min, 40 % after 7 min, maintained for 1 min and re‐establishment of the starting conditions with 100 % A for an additional 2.5 min. The flow rate was 0.35 mL/min, and the column temperature was 35 °C. For MS measurements, an Agilent 6470 Triple Quadrupole mass spectrometer set to dynamic multiple reaction monitoring mode was used. The MS was operated in positive ion mode with the following parameters: skimmer voltage of 15 V, cell accelerator voltage of 5 V, N_2_ gas temperature of 230 °C and N_2_ gas flow of 6 L/min, sheath gas (N_2_) temperature of 400 °C with a flow of 12 L/min, capillary voltage of 2500 V, nozzle voltage of 0 V, and nebulizer at 40 psi.

## Conflict of interest

The authors declare no conflicts of interest.

1

## Supporting information

As a service to our authors and readers, this journal provides supporting information supplied by the authors. Such materials are peer reviewed and may be re‐organized for online delivery, but are not copy‐edited or typeset. Technical support issues arising from supporting information (other than missing files) should be addressed to the authors.

Supporting Information

## Data Availability

Raw data were generated at LAFUGA Genomics, LMU Munich. Derived data supporting the findings of this study are available from the corresponding author [K.J.] on request.
