# Supplementary material for: Opportunities and Challenges to Profile mRNA Modifications in Escherichia coli
Source: Chembiochem. 2022 Jul 29;23(18):e202200270. doi: 10.1002/cbic.202200270 (PMC9542048; doi:10.1002/cbic.202200270)
Supplement: Supplementary file 1 — Supporting Information [file CBIC-23-0-s001.pdf]

ChemBioChem

Supporting Information

Opportunities and Challenges to Profile mRNA Modifications in *Escherichia coli***

Dimitar Plamenov Petrov, Steffen Kaiser, Stefanie Kaiser,* and Kirsten Jung*

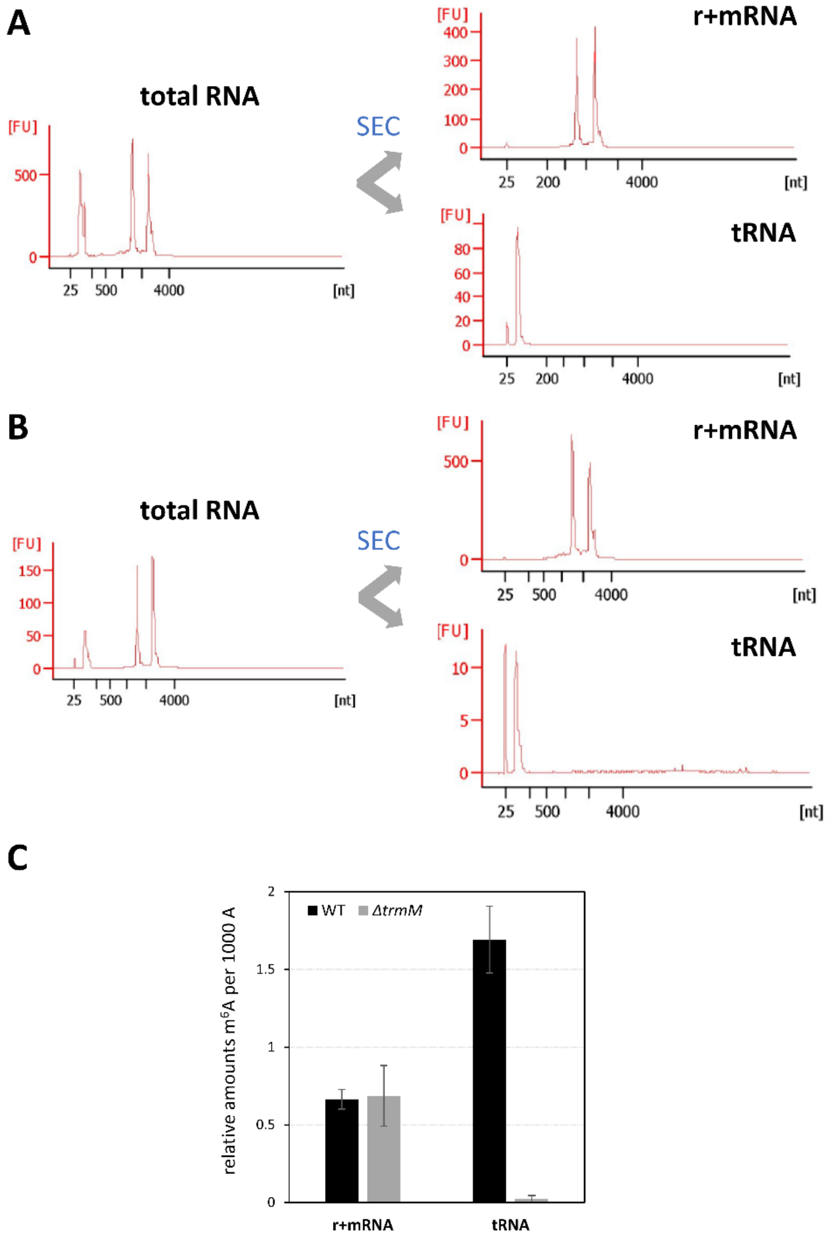

Figure S1. Quality controls for the separation of total RNA from *E. coli* into fractions of tRNA and rRNA+mRNA. Automated chip gel electrophoresis was used to analyze total RNA and the rRNA+mRNA and tRNA fractions after size exclusion chromatography (SEC). RNA was isolated from cells of early-log phase (A) *E. coli* MG1655 (WT) or (B) *E. coli* Δ*trmM* lacking the tRNA-specific m^{6}A methyltransferase TrmM. One representative result is shown for each fraction. (C) LC-MS/MS quantification of the m^{6}A abundance per 1000 A in the rRNA+mRNA and tRNA fractions described in A and B. Results were obtained from at least three biological replicates, error bars represent standard deviation from the means.

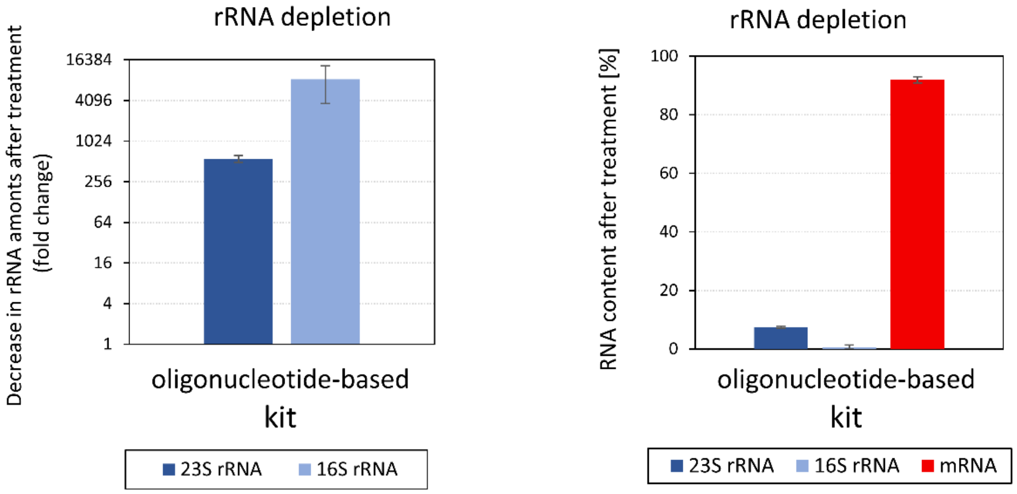

Figure S2. Quality control for the enrichment of mRNA after tRNA removal and rRNA depletion. Left side - RT-qPCR results for the decrease of the rRNA amounts after tRNA removal by SEC and rRNA depletion by using a commercially available oligonucleotide-based kit. The *recA* mRNA was used as internal calibrator for the calculation of the decrease of 23S and 16S rRNA species in the respective samples (see *Material and Methods* for detailed description). Right side - percentage of RNA species in the samples after oligonucleotide-based rRNA-depletion. Values were calculated based on the RT-qPCR results and an assumed ratio of 1% mRNA and 90% rRNA in the total RNA (see *Material and Methods* for detailed description). The results of three biological replicates are presented, each tested in technical triplicates. Error bars represent standard deviation from the means.

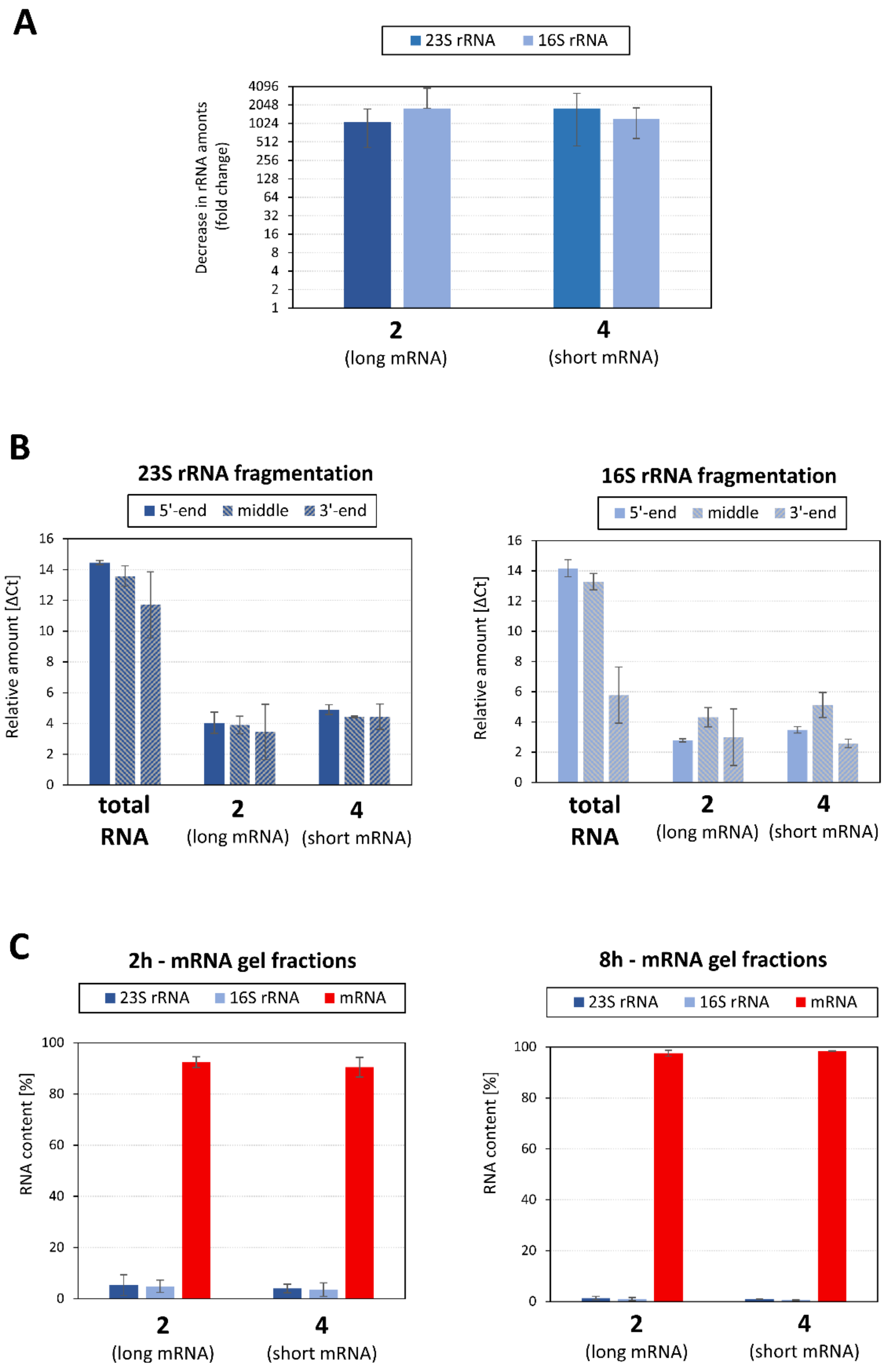

Figure S3. RT-qPCR quality controls for enrichment of mRNA after isolation from denaturing agarose gels. (A) RT-qPCR results for the level of rRNA decrease in fractions 2 and 4. *alaS* and *recA* were used as internal calibrator for the calculation of the decrease of 23S and 16S rRNA species in the respective samples (see *Material and Methods* for detailed description). (B) RT-qPCR results for the relative amounts of 23S rRNA and 16S rRNA fragments covering the 5'-end, middle, and 3'-end sequences found in total RNA or in fractions 2 and 4. The relative amounts were calculated as ΔC_T using *alaS* or *recA* as calibrators. (C) Percentage of RNA species in fractions 2 and 4 extracted after denaturing gel electrophoresis. The time points (2 h and 8 h) refer to the sampling of cells for RNA

preparation from a cultivation (see Figure 3). Values were calculated based on the RT-qPCR results and an assumed ratio of 1% mRNA and 90% rRNA in the total RNA (see *Material and Methods* for detailed description). The results of three biological replicates are presented, each tested in technical triplicates. Error bars represent standard deviation from the means.

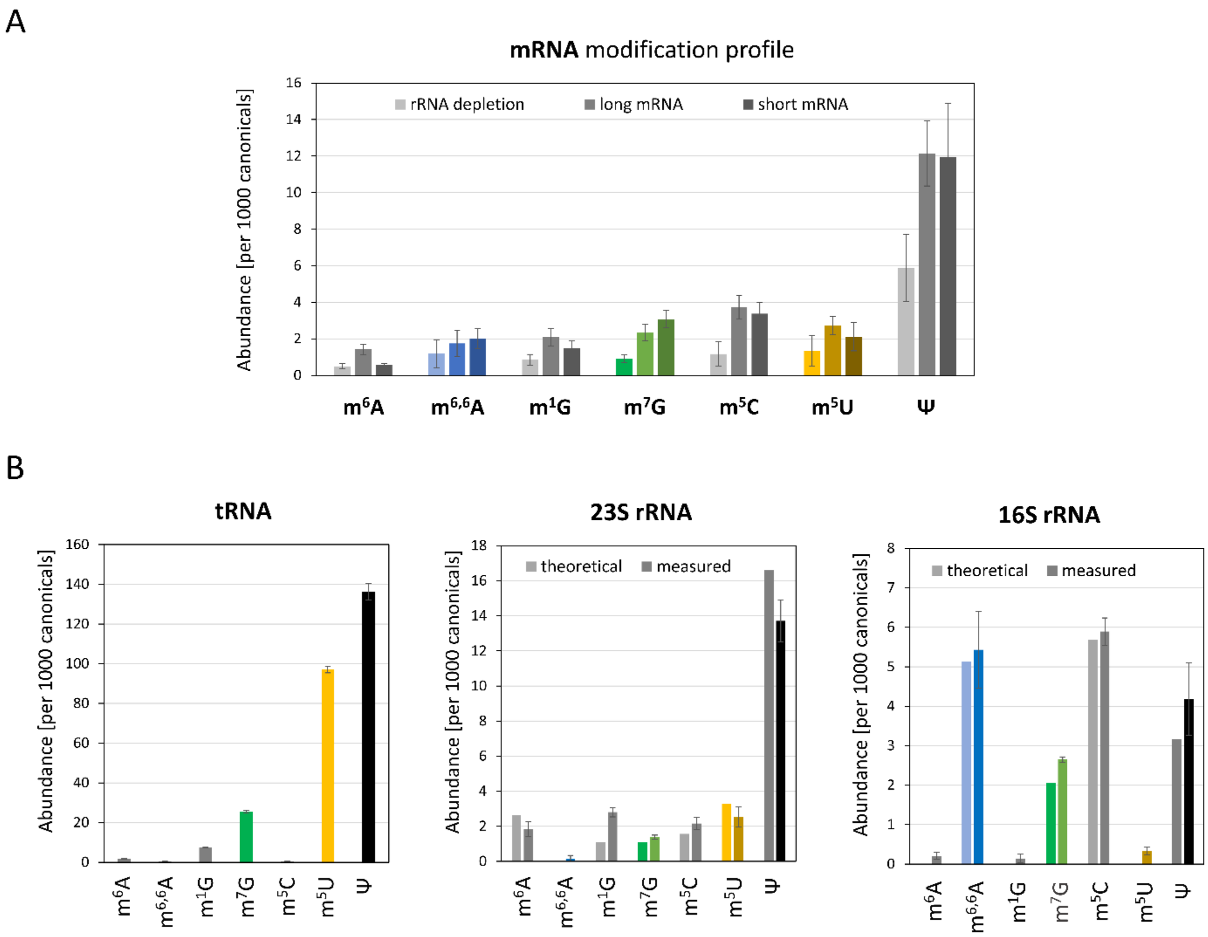

Figure S4. Modification profiles of mRNA, tRNA, and rRNA. (A) Quantitative results of the mRNA modifications are compared after using two different mRNA-enrichment methods: oligonucleotide-based rRNA depletion and denaturing gel electrophoresis [fractions 2 (long mRNAs) and 4 (short mRNAs), data from Figure 3A)]. (B) Quantitative modification profiles of tRNA, 23S rRNA and 16S rRNA. The tRNA fraction was obtained after size exclusion chromatography and quantitively analyzed. Quantitative results of fractions 1 (23S rRNA) and 3 (16S rRNA) (data from Figure 3A) are compared to the expected theoretical abundance. Theoretical values represent the modification abundances in 16S and 23S rRNA, when all known modification sites are modified.

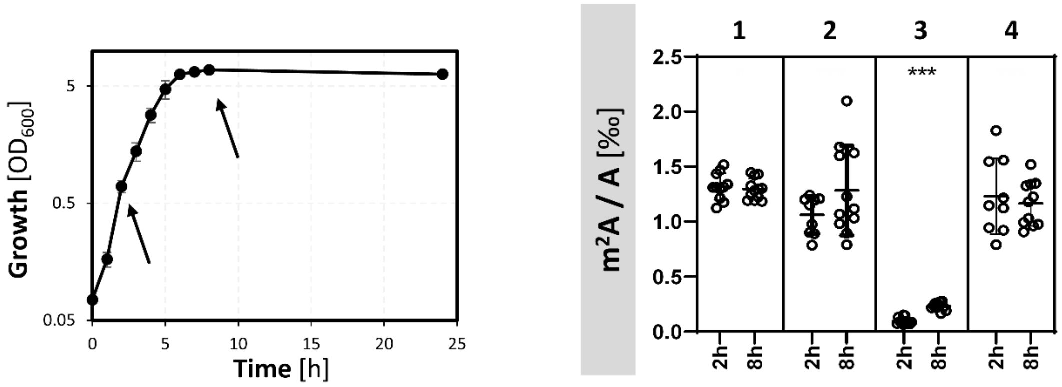

Figure S5. Quantitative analysis of m^2A in RNA of *E. coli* after denaturing agarose gel electrophoresis. RNA was isolated from *E. coli* MG1655 cells after cultivation in complex LB medium under aerobic conditions for 2 h (exponential growth phase) and 8 h (early stationary phase) (left). Time points of RNA extraction are indicated by arrows in the growth curve (left). Following denaturing gel electrophoresis, total RNA was separated in four fractions fraction 1 (23S rRNA), fraction 2 (mRNAs and noncoding RNAs of 1,700 to 2,700 bases), fraction 3 (16S rRNA) and fraction 4 (mRNAs and noncoding RNAs of 400 to 1,100 bases). The fractions were cut from the gel and extracted (right). The relative quantities of m^2A were normalized to the theoretical abundance of m^2A in 23S rRNA (1.31 ‰) assuming that all 23S rRNA molecules are methylated. The results of four biological replicates are presented, each measured in technical triplicates. p-values were calculated by using paired students t-test with *≤0.05, **≤0.01 and ***≤0.001.

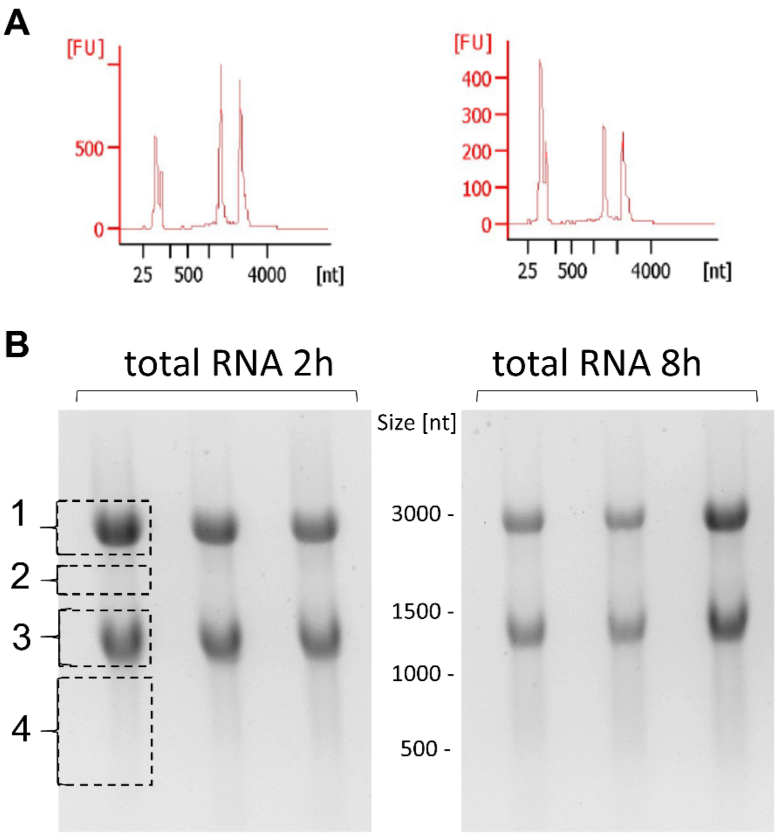

Figure S6. Integrity of total RNA isolated from growing *E. coli* cells. *E. coli* MG1655 was cultivated in LB medium under aerobic conditions at 37°C. At defined time points during growth (2 h and 8 h) cells were harvested, and total RNA was extracted (see Figure 3). (A) Automated chip gel electrophoresis was used to analyze the integrity of each total RNA extraction. (B) After size-dependent separation by a denaturing agarose gel electrophoresis of 10 µg total RNA per lane, fraction 1 (23S rRNA), fraction 2 (mRNAs and noncoding RNAs of 1,700 to 2,700 bases), fraction 3 (16S rRNA) and fraction 4 (mRNAs and noncoding RNAs of 400 to 1,100 bases) were cut from the gel and after rigorous quality controls, hydrolyzed and analyzed by LC-MS/MS.

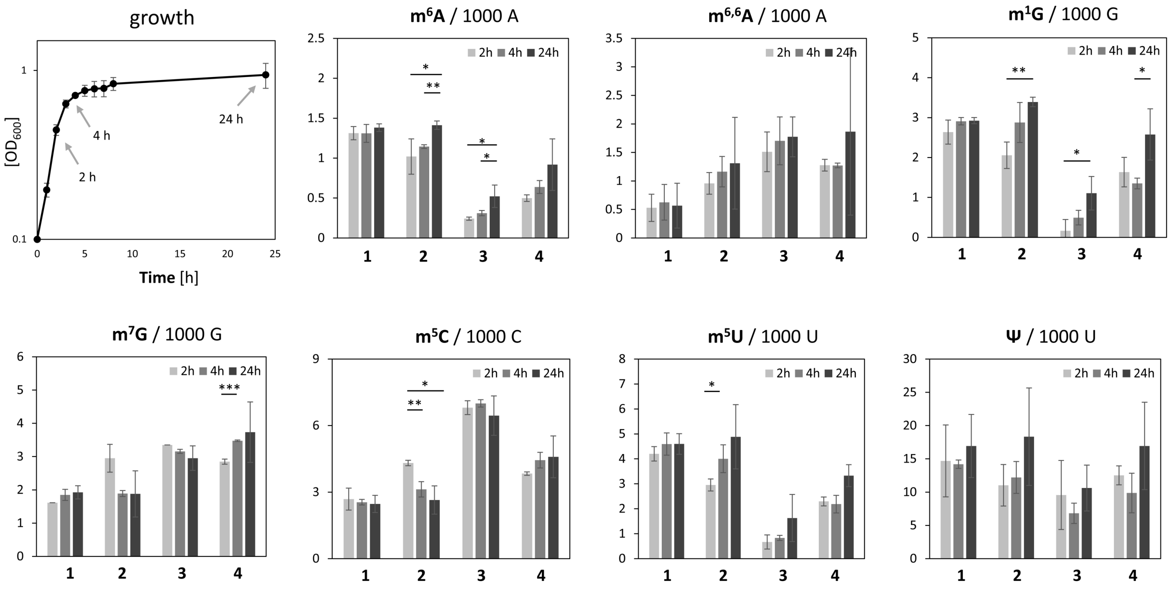

Figure S7. Quantitative analysis of modifications in RNA of microaerobically grown *E. coli* after separation with denaturing gel electrophoresis. *E. coli* MG1655 was grown in LB medium under microaerobic conditions, and samples were taken after 2 h, 4 h and 24 h (arrows in the growth curve). Total RNA was separated using denaturing agarose gel electrophoresis as described in Fig. 2. The fractions were cut from the gel, hydrolyzed and analyzed by LC-MS/MS. The quantities of the modified nucleosides normalized to their respective canonical precursors were obtained by LC-MS/MS. The results of four biological replicates are presented, each measured in technical triplicates. p-values were calculated using paired Student's t-test with *≤0.05, **≤0.01, and ***≤0.001. Error bars represent standard deviation from the means.

Table S1: Oligonucleotides used in this study for the RT-qPCR analysis. All oligonucleotides were produced by Sigma-Aldrich (Missouri, USA).

Primer name	Sequence (5'-->3')
RT-23s_for_front	TAACCGGCGATTTCCGAATG
RT-23s_rev_front	CCAGACGCTTCCACTAACAC
RT-16S_for_front	CGAACGGTAACAGGAAGAAG
RT-16S_rev_front	GCACATCCGATGGCAAGAGG
RT-23S-for_mid	GCAACAAATGCCCTGCTTCC
RT-23S-rev_mid	GTGCCTTCTCCCGAAGTTAC
RT-16S-for_mid	CAGGCGGTTTGTTAAGTCAG
RT-16S-rev_mid	CTTCGCCACCGGTATTCCTC
RT-23s_for_back	CACTGCCCGGTAGCTAAATG
RT-23s_rev_back	ACGCATCGCTGCGCTTACAC
RT-16s_for_back	CGCTAGTAATCGTGGATCAG
RT-16s_rev_back	CCTACGGTTACCTTGTTACG
RT_alaS_for	TCGCTAACCAACAGCTTAACACC
RT_alaS_rev	GCTGAAAGGCGATAGCAATAATC
RT_recA_for	CGGTTCGCTTTCACTGGATATCG
RT_recA_rev	CCTGCAGCGTCAGCGTGGT
